# Trends in stroke incidence, mortality and case fatality rates in Joinville, Brazil: 1995–2006

**DOI:** 10.1136/jnnp.2008.164475

**Published:** 2009-01-15

**Authors:** N L Cabral, A R R Gonçalves, A L Longo, C H C Moro, G Costa, C H Amaral, M V Souza, J Eluf-Neto, L Augusto M Fonseca

**Affiliations:** 1 Department of Medicine of the Universidade da Região de Joinville, Joinville, Brazil and São José Municipal Hospital, Joinville, Brazil; 2 Department of Preventive Medicine Faculdade de Medicina da Universidade de São Paulo, São Paulo, Brazil

## Abstract

**Background:**

Studying stroke rates in a whole community is a rational way to assess the quality of patient care and primary prevention. However, there are few studies of trends in stroke rates worldwide and none in Brazil.

**Objective:**

Established study methods were used to define the rates for first ever stroke in a defined population in Brazil compared with similar data obtained and published in 1995.

**Methods:**

All stroke cases occurring in the city of Joinville during 2005–2006 were prospectively ascertained. Crude incidence and mortality rates were determined, and age adjusted rates and 30 day case fatality were calculated and compared with the 1995 data.

**Results:**

Of the 1323 stroke cases registered, 759 were first ever strokes. The incidence rate per 100 000 was 105.4 (95% CI 98.0 to 113.2), mortality rate was 23.9 (95% CI 20.4 to 27.8) and the 30 day case fatality was 19.1%. Compared with the 1995 data, we found that the incidence had decreased by 27%, mortality decreased by 37% and the 30 day case fatality decreased by 28%.

**Conclusions:**

Using defined criteria we showed that in an industrial southern Brazilian city, stroke rates are similar to those from developed countries. A significant decrease in stroke rates over the past decade was also found, suggesting an improvement in primary prevention and inpatient care of stroke patients in Joinville.

It is currently not clear if stroke rates are changing in different parts of the world. The incidence of total stroke in Latin American and Caribbean countries other than Brazil has been found to be between 135 and 151 per 100 000 population in well designed community based studies.^1^ Only three population based epidemiological studies have been carried out in Brazilian communities where the rates have ranged from 137 to 168 per 100 000 population.^2^^–^4 However, those studies were conducted in different decades and in distinct regions, which complicates the interpretation of trends in rates. There have been no comparative studies in which the same population base was analysed at two different time points.

A recent study demonstrated a decrease in stroke mortality rates over the past two decades for all Brazilian regions,^5^ but it is not clear if this is the result of a decreasing incidence or better health care and management of these patients.

Our aim was to determine the incidence, mortality rates and case fatality of stroke in the city of Joinville and compare our results with a prospective, population based study on first ever stroke carried out in 1995 in the same city using similar methods.^3^

## METHODS

### Study population

In the year 2000 census, the population of the city of Joinville was 429 604 inhabitants, and the projection for the year 2005 was 487 047 inhabitants.^6^ The city has four general hospitals and one public institutional care facility, totalling 840 beds. All of the hospitals, with the exception of the public institutional care facility, have CT services available on a 24 h basis. A pilot study was carried out from August to November of 2004 to test the criteria and feasibility of the study procedures. We named the study Joinville vascular (JOINVASC).

### Method and period of data collection

We obtained information on all cases of first ever stroke occurring in residents of Joinville between 1 January 2005 and 31 December 2006. We used the methodology proposed by Sudlow and Warlow^7^ as well as the Stroke Steps modular programme proposed by the WHO.^8^ In the first step (hospitalised cases), the study nurses registered, on a daily basis, all stroke cases that were confirmed by a neurologist. A neuroradiologist, unaware of the symptoms of the patients, analysed on a weekly basis all brain CT scans and digital angiographies. Every month in the institutional care facility, the electronic records regarding any stroke related diagnoses listed in the 10th revision of the International Classification of Diseases (ICD-10) were reviewed and each medical chart was later discussed with a neurologist (NLC).

In the second step (fatal cases),^8^ we analysed, month by month, all death certificates issued by the Municipal Department of Health. We initially selected all death certificates containing any references to the ICD-10 codes related to stroke (I61 through I69) or any descriptions of cerebrovascular diseases, as well as those listing death as being from an unknown cause (R99). The deaths of patients not identified in the first module were investigated through evaluation of hospital medical charts and, when available, imaging examinations. Sudden deaths occurring within the first 24 h (either at home or in the hospital) not confirmed by brain CT or by a compatible medical history were excluded. This is because these deaths did not always have their underlying causes confirmed by a physician, as were those for which the only previous comorbidity listed on the death certificate was stroke. Finally, some death certificate analyses remained inconclusive even after investigation of the medical charts, or a diagnosis was listed as “undetermined” on the charts. In those cases, the deaths were investigated by contacting the surviving family members. A social worker trained for the task visited the residence of each patient every 2 months. The diagnosis of stroke was considered confirmed if the patient had presented sudden neurological symptoms suggestive of such, with or without cardiovascular risk factors.

In the third step (non-hospitalised alive cases),^8^ we aimed at minimising losses among the patients presenting as mild cases and therefore not admitted to hospital. In the two 24 h emergency care clinics, an automated email notification system was created in order to report all cases in which the patient chart included any of the specific ICD-10 codes for stroke related diseases (I61–I69). However, not all of the patients identified agreed to be admitted. Such patients were offered an appointment as outpatients. Reports were sent to general clinicians working in the public health care system every 3 months. We made annual visits to the departments of ophthalmology, paediatrics and obstetrics. Also, on an annual basis, a neurologist visited every private medical office of the cardiologists, geriatricians and general physicians involved. Every physician received a sticker with the phone number and email address of the researcher nurse which was stuck on the front of the computer monitor as a reminder. We did not monitor patients potentially at risk: those submitted for investigations, such as carotid or limb duplex sonography, or transcranial Doppler; those submitted for angiography (aortic, carotid, coronary or peripheral); and those who underwent surgical interventions, such as carotid, myocardial, aortic or peripheral revascularisation.

### Inclusion and exclusion criteria

We included all cases of residents of Joinville, regardless of age, diagnosed with any type of ischaemic stroke, haemorrhagic stroke or subarachnoid haemorrhage (SAH). We also included residents in the city with confirmed stroke that occurred outside the city limits. Patients experiencing a stroke for the first time, with or without a previous transient ischaemic attack (TIA), were classified as first ever stroke. We excluded cases described as stroke in which the patient died within the first 24 h with no reliable medical record and without a brain CT, patients residing outside the Joinville city limits and patients with subdural, epidural or intracerebral haemorrhage secondary to arteriovenous malformation or tumours.

### Diagnostic criteria

We defined stroke as the presence of signs of sudden focal or global cerebral dysfunction that lasts longer than 24 h without any apparent non-vascular cause.^9^ TIA was defined as a sudden acute loss of cerebral or ocular function, with symptoms lasting less than 24 h, which could be indicative of an embolic or atherothrombotic disease after appropriate investigation.^10^ Infarctions were defined when the CT revealed hypodense brain areas in a topography consistent with the clinical syndrome, and intracerebral haemorrhage was defined when the CT revealed hyperdense brain areas in a topography consistent with the clinical syndrome.^10^ SAH was defined as a sudden, severe headache, occasionally accompanied by loss of consciousness, convulsions or focal neurological signs unrelated to trauma, with the CT revealing hyperdense brain areas in the cisternal or subarachnoid spaces, confirmed either by cerebral digital subtraction angiography or by a non-traumatic lumbar puncture showing an erythrocyte count higher than 2×10^9^/l or a xanthochromic supernatant on CSF analysis.^10^ Cases without brain imaging were classified as undetermined and coded as ischaemic for the rate analyses.

### Routine investigation

After obtaining written informed consent, we obtained demographics and risk factor information, and performed biochemical, electrocardiographic and radiological tests. The attending neurologist then informed the study nurse of the Bamford classification.^11^ The pathophysiological diagnosis of stroke was based on the criteria described in the Trial of ORG 10127 in Acute Stroke Treatment (TOAST) study.^12^ Routine stroke investigation followed the guidelines issued by the Brazilian Society of Cerebrovascular Diseases.^13^

### Outpatient follow-up

All patients were contacted by telephone 30 days after discharge. We registered patient losses resulting from incomplete address, address change or refusal to provide information. Patients who refused to give written informed consent were identified but not followed.

### Statistical analysis

We calculated the 95% confidence interval (CI) assuming a Poisson distribution for the number of events.^14^ Incidence and mortality rates were calculated using intercensal data from 1995 and from the 2005–2006 period as the denominators.^15^ We calculated crude incidence rates and crude mortality rates for the years 2005 and 2006, using the sum of the intercensal population from those years as the denominators and the sum of the cases (deaths) from the same years as the nominators. First ever stroke incidence and mortality rates were age adjusted by the direct method, taking as standards both the 2000 census population of Brazil^15^ and Segi’s “world” population.^16^ We used the χ^2^ test to compare proportions. All tests were two tailed. We created a database using Microsoft Access for the compilation and correlation of data. Statistical analysis was carried out using the Statistical Package for Social Sciences, V.12.0 (SPSS Inc, Chicago, Illinois, USA). The study was approved by the ethics in research committees of the hospitals and universities involved.

## RESULTS

In the 2005–2006 period, we diagnosed 1376 patients with stroke. Of those, eight patients refused to participate in the study and 45 were excluded because of changes in their initial diagnosis of stroke to other diagnoses (29 patients had a definitive diagnosis of seizures, eight patients had a primary or metastatic cerebral tumour and eight had a metabolic encephalopathy). Therefore, the final sample consisted of 1323 patients. Of these, 90 were TIA and 759 were diagnosed with first ever stroke: 610 (80.4%) with ischaemic stroke, 94 (12.4%) with haemorrhagic stroke and 55 (7.3%) with SAH. Of the 759 patients with first ever stroke, 639 (84.2%) were diagnosed in the emergency rooms, 52 (6.8%) through death certificates, 49 (6.5%) in the 24 h emergency care clinics and 19 (2.5%) in private medical offices. No patient was referred from a public outpatient clinic. Of the 52 cases identified based on their death certificates, 24 (46%) were confirmed only after contacting the surviving family members. Three patients who died in the first 24 h without a medical history or brain CT were excluded. Undetermined cases without a brain CT were classified as ischaemic stroke (n = 28).

Of the patients identified with first ever stroke, 385 (51%) were male. Mean age was 64.9 (SD 13.9) years and 707 (93.1%) patients underwent brain CT within 30 days after the event. For patients who did not awake with symptoms (109/759), the mean time from symptom onset to CT was 2.4 (SD 1.2) days. During the study period, 31 (2.3%) patients with stroke underwent intravenous thrombolysis.

The incidence of first ever stroke adjusted to the Brazilian population was 86.6 per 100 000 (95% CI 80.5 to 93.0) and 105.4 per 100 000 (95% CI 98.0 to 113.2) when adjusted to the world population. This is lower than that found 10 years earlier in the same population.^3^ Table 1 shows the incidence rate by sex, adjusted for age in 1995 and in the 2005–2006 period.

**Table 1:** Incidence rates of first ever stroke, by sex, per 100 000 population, Joinville, 1995 and 2005–2006

Age group (years)	Crude incidence (95% CI)		
	Men	Women	Total population
Joinvile 1995			
⩽34	–	–	–
35–44	100.8 (65.8–148.2)	70.8 (42.0–111.9)	85.8 (62.4–115.0)
45–54	211.6 (141.8–304.7)	177.5 (113.8–264.5)	194.7 (145.8–255.1)
55–64	683.3 (514.5–888.3)	312.0 (207.5–452.4)	487.6 (388.6–604.6)
65–74	1079.0 (784.4–1455.9)	698.4 (482.6–963.8)	866.5 (687.1–1074.5)
75–79	2011.2 (1192.6–3177.7)	1031.7 (577.8–1733.3)	1421.0 (972.0–2003.6)
⩾80	1943.2 (1033.8–3322.9)	1310.0 (733.6–2161.5)	1543.6 (1026.5–2238.2)
All	96.4 (83.2–111.6)	70.6 (59.4–83.8)	83.5 (74.6–93.1)
Adjusted Brazil*			113.6 (101.5–126.8)
Adjusted world†			143.7 (128.4–160.3)
JOINVASC 2005–6			
⩽24	–	–	–
25–34	8.1 (3.2–16.7)	10.3 (4,7–19,6)	9.2 (5.3–15.0)
35–44	34.3 (22.4–50.3)	19.4 (10.9–32,0)	26.8 (19.2–36.4)
45–54	120.4 (91.7–155.3)	125.7 (96,6–160,8)	123.0 (102.1–146.9)
55–64	426.7 (347,9–518,0)	202.3 (151,1–265,3)	310.4 (263.3–363.5)
65–74	734.3 (589.7–903.6)	400.8 (319,2–496,9)	523.9 (448.5–608.3)
75–79	865.6 (564.5–1259.4)	707.8 (484,1–999,2)	784.1 (596.9–1011.4)
⩾80	3580.8 (2847.9–4444.7)	2491.2 (2053,1–2995,1)	2856.7 (2469.8–3287.0)
All	80.2 (72.5–88.5)	73.5 (66,2–81,4)	76.9 (71.6–82.6)
Adjusted Brazil*			86.6 (80.5–93.0)
Adjusted world†			105.4 (98,0–113,2)

*Adjusted to the Brazilian population according to the 2000 census.

†Segi’s world population.

**Figure 1:**
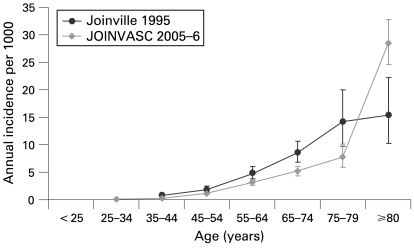
Incidence of first ever stroke, by age group, in 1995 and in the 2005–2006 period. Error bars indicate 95% confidence intervals.

**Figure 2:**
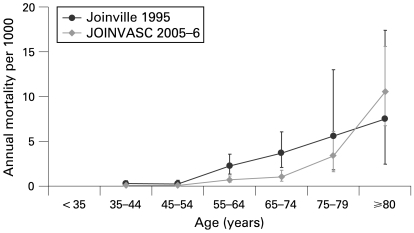
Mortality, by age group, in 1995 and in the 2005–2006 period. Error bars indicate 95% confidence intervals.

The mortality rate adjusted to the Brazilian population was 20.5 per 100 000 (95% CI 17.5 to 23.8) and 23.9 per 100 000 (95% CI 20.4 to 27.8) when adjusted to the world population. This is lower than in 1995.^3^ Table 2 shows the mortality rates by sex in 1995 and in the 2005–2006 period. Figure 1 shows the evolution of age specific incidence rates between 1995 and the 2005–6 period, demonstrating a fall in rates for all age groups, except the very oldest, at the later period studied. The same pattern is seen on fig 2 for mortality rates.

**Table 2:** Stroke mortality rates by sex per 100 000 population, Joinville, 1995 and 2005–2006

Age group (years)	Crude incidence (95% CI)		
	Men	Women	Total population
Joinvile 1995			
⩽24	–	–	–
35–44	27.1 (10.9–55.8)	20.4 (6.6–47.5)	23.4 (12.1–41.0)
45–54	21.9 (4.5–63.9)	29.6 (8.1–75.8)	25.7 (10.3–52.9)
55–64	223.6 (132.6–353.3)	55.7 (18.0–129.8)	135.1 (85.7–202.7)
65–74	367.8 (206.0–606.9)	174.6 (80.0–331.8)	259.9 (166.6–387.3)
75–79	558.7 (181.0–1301.7)	368.5 (119.4–858.6)	391.8 (188.1–720.9)
⩾80	747.4 (242.2–1741.4)	221.1 (45.5–645.6)	441.0 (190.1–868.8)
All	27.6 (20.7–36.2)	16.2 (10.9–23.0)	21.9 (17.5–27.2)
Adjusted Brazil*			29.6 (23.6–36.6)
Adjusted world†			37.5 (29.9–46.5)
JOINVASC 2005–6			
⩽24	–	–	–
25–34	–	1.1 (0.02–6.1)	0.6 (0.01–3.3)
35–44	6.6 (2.1–15.4)	9.1 (2.5–23.3)	5.9 (2.7–11.2)
45–54	6.7 (2.5–14.6)	27.9 (15.2–46.9)	20.2 (12.3–31.1)
55–64	71.1 (41.4–113.8)	35.0 (16.0–66.5)	52.4 (34.2–77.0)
65–74	99.0 (51.2–173.3)	96.6 (59.0–148.8)	97.5 (66.7–137.5)
75–79	332.9 (159.8–612.5)	154.8 (62.1–318.9)	225.9 (131.5–361.4)
⩾80	1048.0 (671,8–1561.5)	903.9 (648.7–1126.2)	952.2 (734.9–1213.7)
All	15.1 (11.9–19.0)	19.2 (15.6–23.4)	17.2 (14.7–20.0)
Adjusted Brazil*			20.5 (17.5–23.8)
Adjusted world†			23.9 (20.4–27.8)

*Adjusted to Brazilian population according to the 2000 census.

†Segi’s world population.

Table 3 shows the differences between the two periods in terms of the incidence and mortality rates of first ever stroke, by sex and age group. Incidence adjusted for age fell by 26% (p = 0.02) in males and by 20% (p = 0.21) in females although only among individuals younger than 75 years of age (41%; p = 0.003).

**Table 3:** Standardised mortality and incidence rates of first ever stroke, by sex and age, per 100 000 population and 30 day case fatality rate in Joinville, 1995 and 2005–2006

	1995*	2005–6†	Relative incidence	p Value‡
Mortality	1995*	2005–6†	Relative mortality	p Value‡
Incidence				
Men	145.1 (124.9–167.6)	108.6 (98.1–119.9)	0.74 (0.59–0.96)	0.02
Women	88.3 (74.0–104.5)	71.5 (64.4–79.2)	0.80 (0.60–1.12)	0.21
Age <75 years	83.9 (74.0–94.7)	50.3 (46.0–54.9)	0.59 (0.42–0.84)	0.003
Age ⩾75 years	29.6 (22.6–38.1)	36.4 (32.1–41.2)	1.22 (0.74–1.95)	0.46
All	113.6 (101.5–126.8)§	86.6 (80.5–93.0)§	0.76 (0.58–1.01)	0.06
	143.7 (128.4–160.3)¶	105.4 (98.0–113.2)¶	0.73 (0.57–0.94)	0.01
Men	43.0 (32.2–56.2)	22.5 (17.7–28.2)	0.52 (0.32–0.89)	0.01
Women	19.8 (13.5–28.1)	19.4 (15.7–23.4)	0.97 (0.51–1.78)	0.87
Age <75 years	21.6 (16.7–27.5)	8.7 (7.0–10.7)	0.40 (0.19–0.89)	0.02
Age ⩾75 years	8.3 (4.9–13.1)	11.8 (9.4–14.6)	1.42 (0.61–3.67)	0.37
All	29.6 (23.6–36.6)§	20.5 (17.5–23.8)§	0.69 (0.40–1.22)	0.21
	37.5 (29.9–46.5)¶	23.9 (20.4–27.8)¶	0.63 (038–1.05)	<0.01
Case fatality	26.6 (84/320)	19.1 (145/759)	28	0.009

*Brazilian population according to the intercensal projection for 1995.

†Intercensal projection for the 2005–2006 period.

‡For primary analysis, 1995 versus 2005–2006.

§Adjusted to Brazilian population census 2000.

¶Adjusted to world Segi's population.

There was also a decrease in the mortality adjusted for age, although it was much more pronounced in males (48%) than in females (3%).

The case fatality was 19.1% (145/759) in the 2005–2006 period, which is also lower than that found in 1995 (26.6% (84/320)).^3^ Therefore, over a span of approximately 10 years, the incidence fell by 27% and mortality fell by 37%. The 30 day case fatality decreased by 28.2% during the period (from 26.6% to 7.5%).

## DISCUSSION

We have shown that, over an interval of nearly 10 years, there was a significant decrease in the incidence, mortality and 30 day case fatality of first ever stroke in Joinville, Brazil. Mortality adjusted for age decreased by 37%. A recent study on mortality corroborates these findings.^5^ André *et al*, using official Brazilian Ministry of Health data, reported that the adjusted mortality rates decreased by 40% (68.2 vs 40.9 per 100 000 population) from 1980 to 2000, while in the Southern region of Brazil, where Joinville is located, mortality decreased by 44% (79.9/44.0).^5^

What could have caused the 37% reduction in stroke mortality in Joinville over those 10 years? Classically, mortality is considered to be directly influenced by incidence and case fatality rates.^17^ The Monitoring Trends and Determinants in Cardiovascular Disease (MONICA) project, which included no Latin American countries, showed that in the populations presenting declining mortality rates, one-third of the decrease could be attributed to incidence and two-thirds to case fatality.^17^ In Joinville, stroke incidence and case fatality significantly decreased over the past 10 years. However, what led case fatality to decrease by 28.2% and the incidence adjusted for age for the world population to decrease by 27%?

Currently, Joinville ranks 13th among Brazilian cities in terms of its human development index, which increased from 0.779 in 1991 to 0.857 in 2000.^18^ The increase in socioeconomic conditions in the city might have influenced primary prevention and, subsequently, the decrease in incidence.^17^

In our study, however, incidence rates increased among individuals over 75 years of age. We believe this was caused by the increase in life expectancy, with a subsequent increase in the absolute number of older people. Over the past 20 years, the elderly population of Joinville has increased by 151%, from 11 263 in 1980 to 28 236 in 2000.^15^ ^19^ In 1991, life expectancy was 70.6 years compared with 76.6 years in 2000.^19^

Another reason for the decrease in mortality was the 28% decrease in 30 day case fatality. In one review article on the effectiveness of stroke treatment, it was reported that the public health care measures that had a higher impact on stroke prevention were the presence of stroke units, followed by the routine use of aspirin and thrombolysis.^20^ The largest hospital in Joinville has had a stroke unit since 1997.^21^ Since 2002, intravenous thrombolysis has been used with increasing frequency in most hospitals in the city^22^ ^23^ although this is unlikely to alter case fatality^24^ In the JOINVASC study, 31 (2.3%) of the patients received thrombolytic treatment. In one study on hospitalisations due to cerebral infarction occurring in the USA between 1999 and 2004, it was reported that only 1.12% of the patients received thrombolytic treatment.^25^ Another aspect that influences case fatality is the severity of the events. Considering 13 of the 15 population based studies on stroke selected by Feigin *et al*, mean case fatality was 22.9%.^26^ Case fatality for first ever stroke, regardless of subtype, was 19.1% in the JOINVASC study and 23% in the Proyecto Investigación de Stroke en Chile: Iquique Stroke (PISCIS) study. Moreover, comparing the case fatality rates by subtypes from both studies we can see that the Joinville’s rates were always lower, although CIs overlapped.^27^ Therefore, we assume that the decrease in case fatality in the present study was not caused by variations in the severity of the events in our population but rather by the quality of inhospital care.^17^

Can the results obtained in the two periods be compared methodologically? Studies on incidence trends over time can be impaired by changes in the techniques of investigation and diagnosis.^28^ In order to guarantee comparability, we used the same methodology in 1995 and in the 2005–2006 period.^3^ In 1995, 98% of the study sample underwent brain CT, as did 93.1% (707/759) of the sample in the 2005–2006 period. The decrease in brain CT ascertainment on this last period could be explained by the inclusion of a public institutional care facility, where brain CT was not available, and also by the active search of patients performed by the social worker. The medical staff were essentially the same. In a recent review of population based studies carried out in Latin America and the Caribbean, the Joinville study in 1995 was reported to be semi-populational based as survivors not hospitalised (third WHO module) were not included in the sample.^1^ ^3^ In this 2005–2006 study, we made an effort to improve the recruitment of all stroke cases. The public outpatient clinic system in Joinville is not totally computerised and electronic medical charts are still unavailable, which makes data recovery difficult. However, in order to improve recruitment of patients who had not been hospitalised (hot pursuit), we used three additional resources: stickers stuck to the computer monitors in the medical offices of non-neurologist physicians; an email notification system in the 24 h emergency care clinics; and reports communicating the progress of the study to the physicians in the city every 6 months. The use of the first two resources, as case recruitment sources, resulted in a 2.5% increase in the total sample. The hot pursuit provided a 5.5% increase in the sample older than 60 years of age in the OXVASC.^29^ This is the main weakness of our study. Therefore, if we consider the lower recruitment of non-fatal cases in 1995,^3^ ^30^ we believe that the 27% decrease in the adjusted incidence in the past 10 years might be underestimated. In the same way as the denominator will rise in the 2005–6 period, the decrease in case fatality rates found could be overestimated.^30^

The percentage variations in the incidence, mortality rates and case fatality of first ever stroke over time in Joinville were similar to those found in the cities of Espoo-Kuaniainen (Finland), Oyabe (Japan), Perth (Australia), Novosibirski (Russia), Auckland (New Zealand) and Oxfordshire (UK).^26^

In conclusion, there was a consistent decrease in morbidity, mortality rates and case fatality of first ever stroke in the city of Joinville, Brazil, from 1995 to 2006. The findings regarding incidence might have been influenced by the socioeconomic characteristics of the city of Joinville. Extrapolation of these results to other populations should be considered with caution, especially in a country as heterogeneous as Brazil. Although we cannot prove that the decrease in the incidence resulted directly from the control of risk factors for atherosclerosis, the size of the changes suggests advances in the quality of primary prevention.

## References

[1] Lavados PM Hennis AJ Fernandes JG Stroke epidemiology, prevention and management strategies at a regional level: Latin America and the Caribbean. Lancet Neurol 2007;6:362–72 17362840 10.1016/S1474-4422(07)70003-0

[2] Lessa I Bastos CA Epidemiology of cerebrovascular accidents of Salvador, Bahia, Brazil. Bull Pan Am Health Organ 1983;17:292–303 6652322

[3] Cabral NL Longo AL Moro CHC Epidemiologia dos acidentes cerebrovasculares em Joinville, Brasil. Arq Neuropsiquiatr 1997;55:357–63 9629349 10.1590/s0004-282x1997000300002

[4] Minelli C Fen LF Minelli DPC Stroke incidence, prognosis, 30-day, and 1-year case fatality rates in Matao, Brazil. Stroke 2007;38:2906–11 17916767 10.1161/STROKEAHA.107.484139

[5] André C Curioni CC da Cunha CB Progressive decline in stroke mortality in Brazil From 1980 to 1982, 1990 to 1992, and 2000 to 2002. Stroke 2006;37:2784–9 17008629 10.1161/01.STR.0000244768.46566.73

[6] Instituto Brasileiro de Geografia e Estatística IBGE.. http://www.ibge.gov.br/home/estatistica/populacao/censo2000/defaulttab_munic.shtm.

[7] Sudlow CL Warlow CP Comparing stroke incidence worldwide: what makes studies comparable? Stroke 1996;27:550–58 8610328 10.1161/01.str.27.3.550

[8] The WHO STEPwise approach to Stroke Surveillance Overview and Manual (V.2.0). Noncommunicable Diseases and Mental Health. World Health Organization. Disponível em.. www.who.int/entity/ncd_surveillance/steps/en.

[9] Aho K Harmsem P Hatano S Cerebrovascular diseases in the community: results of a WHO collaborative study. Bull World Health Organ 1980;58:113–30 6966542 PMC2395897

[10] Hankey GJ Warlow CP Transient ischaemic attack of the brain and eye London: WB Saunders, 1994

[11] Bamford P Sandercock M Dennis J Classification and natural history of clinically identifiable subtypes of cerebral infarction. Lancet 1991;337:1521–6 1675378 10.1016/0140-6736(91)93206-o

[12] Adams HP Jr Bendixen BH Kappelle LJ and the TOAST Investigators. Classification of subtype of acute ischemic stroke. Definitions for use in a multicenter clinical trial. Stroke 1993;24:35–4 7678184 10.1161/01.str.24.1.35

[13] Sociedade Brasileira de Doenças Cerebrovasculares Brazilian guideline for the management of acute stroke. Arq Neuropsiquiatr 2001;59:972–8011733849

[14] Keyfitz N Sampling variance of standardized mortality rates *Hum Biol* 1966;38:309–17 5977534

[15] Ministério da Saúde População residente por sexo segundo faixa etária- Municipio: Joinville.. http//:www.tabnet.datasus.gov.br/cgi/deftohtm.exe?ibe/cnv/popsc.def.

[16] Ahmad OB, Boschi-Pinto C, Murray CJL http://www.who.int/healthinfo/paper31.pdf.

[17] Asplund K What MONICA told us about stroke? Lancet Neurol 2005;4:64–8 15620858 10.1016/S1474-4422(04)00967-6

[18] Programa das Nações Unidas para o Desenvolvimento Atlas do Desenvolvimento Humano. Ranking do IDH-M dos municípios do Brasil. Disponível em.. http://www.pnud.org.br/atlas/tabelas/index.php.

[19] Mastroeini MF Erzinger GS Mastroeini SSBS Demographic profile of the elderly in the city of Joinville, Santa Catarina: a household survey. Rev Bras Epidemiol 2007;10:190–201

[20] Hankey GJ Warlow CP Treatment and secondary prevention of stroke: evidence, costs and effects on individuals and populations. Lancet 1999;354:1457–63 10543686 10.1016/S0140-6736(99)04407-4

[21] Cabral NL Moro C Silva GR Study comparing the stroke unit outcome and conventional ward treatment. Arq Neuropsiq 2003;61:188–93 10.1590/s0004-282x2003000200006 12806495

[22] Massaro AR Stroke in Brazil: a South America perspective. Int J Stroke 2006;1:113–15 18706058 10.1111/j.1747-4949.2006.00029.x

[23] Pandian JD Padma V Vijaya P Stroke and thrombolysis in developing countries. Int J Stroke 2007;2:17–26 18705983 10.1111/j.1747-4949.2007.00089.x

[24] Wardlaw JM Zoppo G Yamaguchi T Thrombolysis for acute ischaemic stroke. Cochrane Database Syst Rev 2003;3:CD000213 12917889 10.1002/14651858.CD000213

[25] Schumacher HC Bateman BT Boden-Albala B Use of thrombolysis in acute ischemic stroke: analysis of the National Inpatient Sample 1999 to 2004. Ann Emerg Med 2007;50:99–107 17478010 10.1016/j.annemergmed.2007.01.021

[26] Feigin VL Lawes CM Bennett DA Stroke epidemiology: a review of population-based studies of incidence, prevalence, and case fatality in the late 20th century. Lancet Neurol 2003;2:43–53 12849300 10.1016/s1474-4422(03)00266-7

[27] Lavados PM Sacks C Prina L Incidence, 30 day case-fatality rate and prognosis of stroke in Iquique, Chile: a 2 year community-based prospective study (PISCIS project). Lancet 2005;365:2206–15 15978929 10.1016/S0140-6736(05)66779-7

[28] Haan R Limburg M Bossuyt P The Clinical Meaning of Rankin Handicap’ Grades After Stroke. Stroke 1995;26:2027–30 7482643 10.1161/01.str.26.11.2027

[29] Rothwell PM Coull AJ Gilles MF Change in stroke incidence, mortality, case-fatality and risk factors in Oxfordshire, UK from 1981 to 2004 (Oxford Vascular Study). Lancet 2004;363:1925–33 15194251 10.1016/S0140-6736(04)16405-2

[30] Rothman KJ Epidemiology an introduction Oxford: Oxford University Press, 2002

